# THE PRESHAVING PROTOCOL IN BODY HAIR-TO-SCALP TRANSPLANT TO IDENTIFY HAIR IN ANAGEN PHASE

**DOI:** 10.4103/0019-5154.60353

**Published:** 2010

**Authors:** Arvind Poswal

**Affiliations:** *From Dr. A's Clinic, A-9, 1^st^ Floor, Chitranjan Park, New Delhi - 110 019, India.*

**Keywords:** *Androgenic alopecia*, *follicular unit hair transplant*, *anagen*, *telogen*, *body hair*

## Abstract

**Introduction:**

The use of body donor hair for transplanting to the bald scalp is termed body hair transplant. In recent times, robust body hair has been used as an adjunct to scalp donor hair to augment the donor hair supply. A large percentage of body hair are in telogen and, as single hair units.

**Aims:**

To devise a non invasive protocol to identify the body donor hair in anagen phase prior to extraction.

**Materials and Methods:**

Hairs are shaved flush with the skin, four days prior to extraction. On fourth day, the actively growing hair follicles as well as non growing hairs were extracted and phase of hair growth determined. Results: Nineteen out 22 extracted hair follicles in nongrowing phase were found to be the telogen phase.

**Conclusion:**

Preshaving the body donor areas is a simple non invasive method of isolating the hair in anagen phase.

## Introduction

Follicular unit hair transplant is the most common surgical treatment for Androgenic alopecia or pattern hair loss.[1] For patients with extensive hair loss (Norwood 5 and above),[2] scalp donor hair alone is not sufficient to give good cosmetic improvement. In such patients, robust body hair donor hair has been used as an adjunct to the scalp donor hair.[3] The use of body donor hair for transplanting to the bald scalp is termed body hair transplant (BHT).[3]

Unlike scalp donor hair follicular units:

1. A higher percentage of body hair grows as single hair follicular units.
2. A high percentage (40 to70%) of the body hair is in resting telogen phase (not exogen) at any particular time.

The dermal components of the hair (the dermal papilla) are attenuated during telogen, and more prone to damage during individual follicular extraction. Therefore, only body donor hair follicles in the active anagen phase are preferred for transplant. Since all the telogen hair do not shed, and may be visible in the body donor area, it is necessary to employ a non invasive method to identify the hair in anagen phase, before extraction.

The preshaving protocol is a simple, non invasive way to identify body donor hair in anagen phase.

## Methods and Discussion

**Figure 1:**
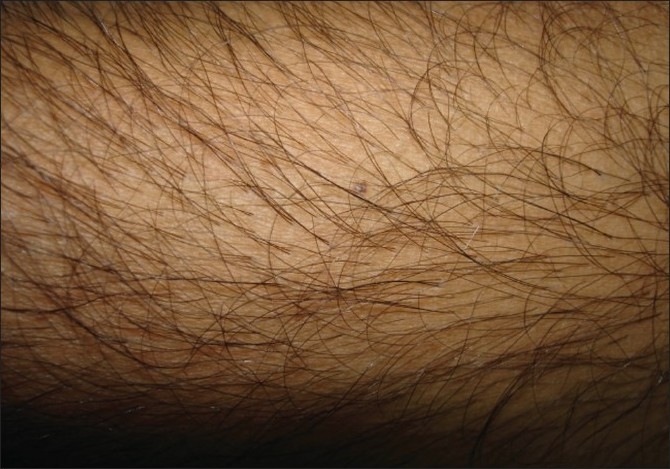
Before

**Figure 2:**
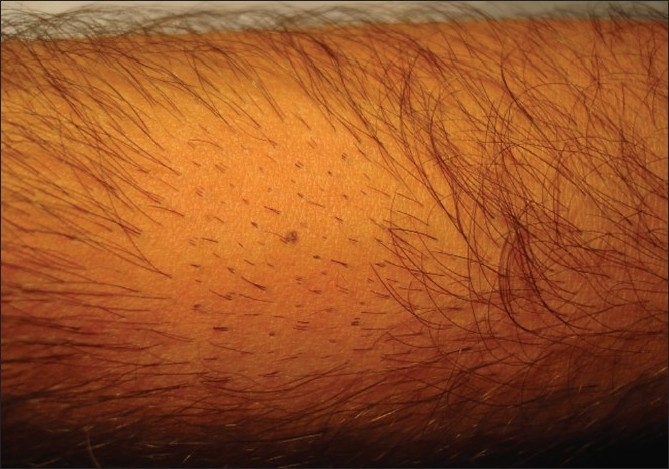
Hair trimmed - Not shaved

**Figure 3:**
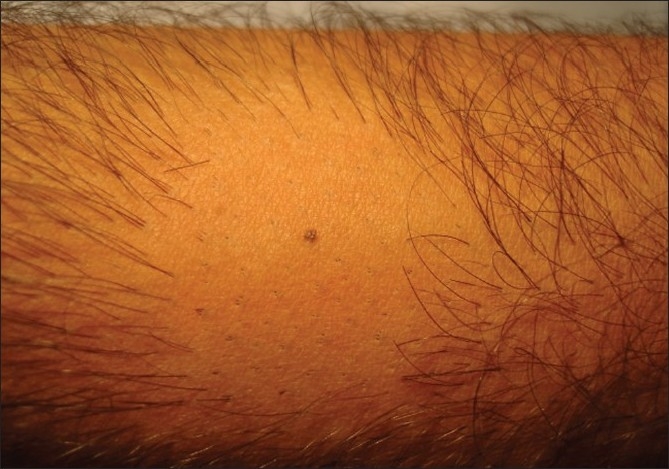
Hair wet shaved with razor

**Figure 4:**
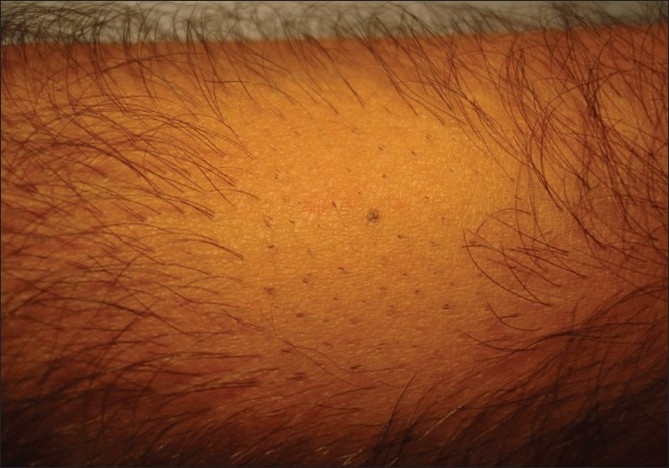
One day after wet shaving

**Figure 5:**
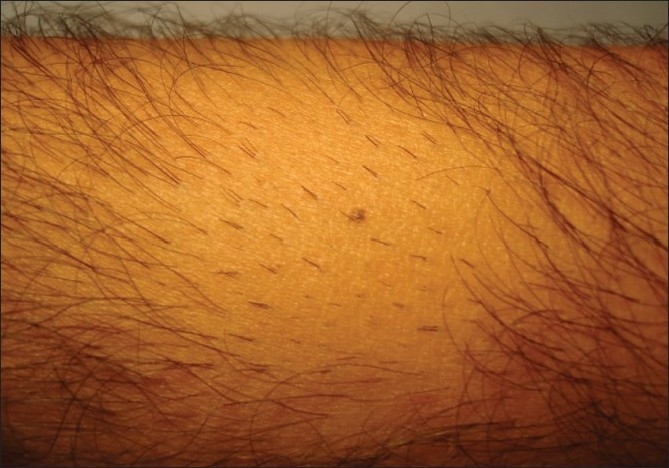
Four days after wet shaving

**Figure 6:**
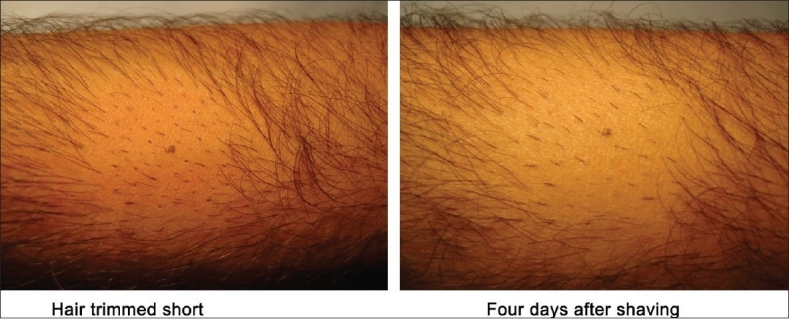
Comparative picture

The patient shaves the body donor hair with a razor. The hairs are shaved flush with the skin, four days prior to extraction. At the end of three to four days, the actively growing hair is easy to identify due to their increased length. The accompanying picture sequence illustrates this method - Figure 1 is taken before any shaving or trimming of the hair on the forearm. The mole is the reference point in all the pictures. Figure 2 shows the area with the hair trimmed to 1 to 2 mm. At this length, it is not feasible to distinguish the hair in anagen from those in telogen. Figure 3 shows the area immediately after wet shaving flush to the skin surface, Figures 4 and 5 show the same area, one and four days after shaving. As can be seen in Figure 6, showing comparison, there are more visible, long hair after just trimming compared to four days after shaving.

The non growing hair (catagen + telogen) did not show any increase in length after being wet shaved. Thus, preshaving the body donor area three to four days prior to extraction of the body donor hair, is a simple method of pinpointing the hair in anagen. The actively growing hair follicles are then teased out of the skin with help of a 20 gauge hypodermic needle under local anesthesia. In a volunteer patient, when the preshaving protocol was used and non growing hair follicles were extracted, 19 out 22 extracted hair follicles were found to be the telogen phase. Additional points to consider in the preshaving protocol include (1) Incidence and treatment of folliculitis: Wet shaving the proposed body donor areas causes mild folliculitis in some patients. Folliculitis is found to be more common in axillary and pubic areas. It is also found to occur in patients with curly hair. Teasing out the ingrown hair followed by application of Mupirocin ointment twice a day for three days resolves the condition; (2) The effect of transplanted body hair growth cycles-40 to 70% of the body hair are in telogen at any particular time. In addition, their duration of anagen is much shorter (12 to 16 weeks) as compared to the scalp donor hair (2 to 5 years). Preshaving pinpoints body donor hair whose growth cycles are more closely synchronized. Transplanting these hair results in a closer synchronization of the growth and shedding phase of the transplanted body hair in the initial growth cycles. However, over a two year period, as the hair go through repeated growth cycles, the growth phase of these transplanted body hair lose their synchronicity.

## Conclusion

Compared to the scalp donor hair, large percentage of body hair are in telogen at any given time. Preshaving the body donor hair three to four days prior to extraction is an easy way to identify hair in anagen phase that can then be used for transplanting.
